# A Rare Diagnosis of Aseptic Hypertrophic Pachymeningitis: A Case of Mycobacterial Tuberculosis Origin

**DOI:** 10.7759/cureus.45973

**Published:** 2023-09-26

**Authors:** Rahul Gunde, Jayashankar CA, Hiba Salam, Ganaraja V Harikrishna, Suresha Kodapala

**Affiliations:** 1 Neurology, Vydehi Institute of Medical Sciences and Research Centre, Bangalore, IND; 2 Internal Medicine, Vydehi Institute of Medical Sciences and Research Centre, Bangalore, IND

**Keywords:** extrapulmonary tuberculosis (eptb), tb meningitis, central nervous system tuberculosis, hypertrophic pachymeningitis, tubercular meningitis

## Abstract

Tubercular meningitis is a rare yet devastating type of extrapulmonary tuberculosis (TB) posing great diagnostic challenges due to the nonspecific clinical presentation of the patients. Here, we present a rare diagnosis of hypertrophic pachymeningitis due to *Mycobacterium tuberculosis*. A 36-year-old male presented with a history of headaches and giddiness for one month. Neurological examination revealed hypo-reflexive triceps and ankle reflexes. Routine blood tests and autoimmune workup were normal. Brain MRI with contrast revealed diffuse dural thickening, focal leptomeningeal enhancement in the right temporal sulci, and enhancement in both the frontal and parietal convexity and the falx cerebri and along the tentorium cerebelli. Cerebrospinal fluid (CSF) analysis revealed elevated proteins, suggestive of aseptic meningitis. Meningeal biopsy revealed a chronic ill-formed granulomatous inflammatory lesion with occasional acid-fast bacilli, consistent with tubercular pachymeningitis. The patient was administered intravenous (IV) methylprednisolone for five days, following which the symptoms subsided. He was advised tablet prednisolone on discharge, and immunomodulation with rituximab was recommended as outpatient treatment. Hypertrophic pachymeningitis is a rare diagnosis characterized by the inflammation and fibrosis of the dura matter due to a diverse etiology. Tubercular etiology must be considered when the routine laboratory tests are negative, and the diagnosis should be confirmed by meningeal biopsy. The treatment of the underlying cause and corticosteroids remain the mainstay management of hypertrophic pachymeningitis. Hence, mycobacterial tuberculosis should be considered as a possible differential diagnosis while evaluating hypertrophic pachymeningitis, especially when the routine laboratory tests and immunological workup are negative.

## Introduction

Tuberculosis (TB) is a major public health burden across the globe, with high mortality and morbidity rates [1,2]. It remains the single most common cause of death due to an infectious disease [2]. Tubercular meningitis, a rare yet devastating type of extrapulmonary tuberculosis, poses great diagnostic challenges due to its nonspecific clinical presentation [1-3]. Hence, the diagnosis of tubercular meningitis requires a high degree of clinical suspicion [4]. Hypertrophic pachymeningitis is a rare disorder characterized by the inflammation and fibrosis of the dura mater, leptomeninges, and tentorium [4,5]. The various etiologies of pachymeningitis include infection, trauma, autoimmune diseases, connective tissue diseases, sarcoidosis, and malignancy, with the most common cause being idiopathic [4,5]. Although tuberculosis is a rare cause of pachymeningitis, and is often difficult to diagnose, requiring meningeal biopsy, it must be considered while evaluating the differential diagnosis of pachymeningitis as it is treatable with antitubercular treatment [5,6]. Here, we present a case of aseptic hypertrophic pachymeningitis due to *Mycobacterium tuberculosis* diagnosed by meningeal biopsy.

## Case presentation

A 36-year-old male presented to the neurology outpatient department (OPD) with a one-month history of persistent headaches in the bitemporal and occipital region, which were exacerbated by exposure to sunlight. The headaches were often accompanied by giddiness. During these episodes of giddiness, he also develops weakness and pain in the lower limbs, prompting him to take support and sit down, due to his tendency to fall during these episodes. He also reports a 15-day history of lower back pain, which is not associated with any features of radiculopathy. He also complains of sensory symptoms such as pins and needles sensation, tingling, and numbness in both lower limbs from the ankle to the feet. He has no history of any recent loss of weight, loss of appetite, or cough. His personal history is also significant for tobacco and alcohol consumption.

On examination, the patient was alert and oriented to time, place, and person. His vitals were stable with a regular heart rate of 80 beats per minute and blood pressure of 120/80 mm Hg. There was no lymphadenopathy on thorough physical examination. Neurological examination revealed normal higher mental functions with no motor, sensory, or cranial nerve deficits; meningeal signs; and cerebellar signs except hypo-reflexive triceps and ankle reflexes.

His routine laboratory investigations, complete hemogram, liver and kidney function tests, blood glucose, coagulation profile, and serum electrolytes, were within normal ranges (Table 1). Erythrocyte sedimentation rate (ESR) was 20, and serological markers for human immunodeficiency virus (HIV), hepatitis B, hepatitis C, and syphilis were negative (Table 1).

**Table 1:** Investigations of the patient with hypertrophic pachymeningitis

Investigation	Patient values	Normal values
White blood cell count (10^9^/L)	6.9	4-10
Hemoglobin (g/dL)	14.1	13-17
Platelet count (10^9^/L)	194	150-400
Aspartate transaminase (units/L)	26	5-30
Alanine transaminase (units/L)	29	5-30
Total protein (g/dL)	7.86	6-8
Albumin (mg/dL)	4.50	3.5-5
Alkaline phosphatase (units/L)	71	50-100
Globulin (g/dL)	3.36	2.3-3.4
Lactate dehydrogenase (units/L)	243	50-150
Glycosylated hemoglobin (HbA1C) (%)	6.3	4-6
Thyroid-stimulating hormone (µU/mL)	2.98	0.5-5
Serum creatinine (mg/dL)	1.9	0.8-1.3
Serum blood urea nitrogen (mg/dL)	18	8-21
Blood glucose level (mg/dL)	120	65-110
Serum C-reactive protein (mg/L)	Negative	<5
Erythrocyte sedimentation rate (ESR) (mm/hour)	17	<18
Serum cortisol (μg/dL)	7.10	5-23
Anti-cyclic citrullinated peptide (anti-CCP) antibody	Negative	Negative
Prothrombin time (seconds)	11.7	11-14
International normalized ratio (INR)	1.05	0.9-1.2
Activated partial thromboplastin time (seconds)	24.9	20-40
Serum sodium (mEq/L)	138	135-145
Serum potassium (mEq/L)	4.2	3.5-5
Human immunodeficiency virus (HIV)	Negative	Negative
Hepatitis B	Negative	Negative
Hepatitis C	Negative	Negative
Syphilis, rapid plasma reagin	Negative	Negative
Antinuclear antibody (ANA)	Negative	Negative
Angiotensin-converting enzyme	25	23-57 U/L
Antineutrophil cytoplasmic antibodies (ANCA)	Negative	Negative
Vitamin B12 (pg/mL)	262	190-950
Immunoglobulin G4	Negative	Negative
Cerebrospinal fluid (CSF) meningoencephalitis panel	Negative	Negative
CSF GeneXpert	Negative	Negative
CSF protein (mg/dL)	195.5	20-40
CSF glucose (mg/dL)	76.03	50-75
CSF white blood cell count (cells/mm^3^)	6	0-5 cells/mm^3^
CSF cell type	100% lymphocytes	
CSF Gram stain	Negative	Negative
CSF Ziehl-Neelsen stain	Negative	Negative
CSF opening pressure (centimeters of water)	12	<20

**Figure 1:**
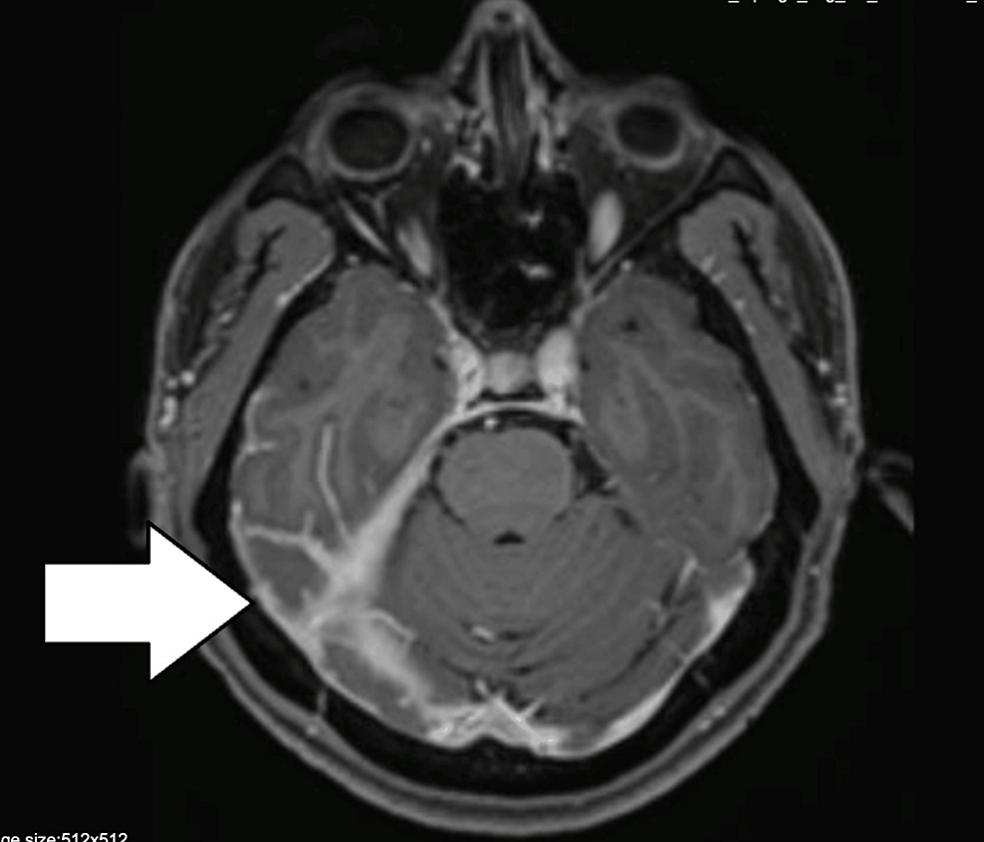
MRI of the brain: T1 axial contrast showing contrast enhancement in the right tentorium cerebelli

**Figure 2:**
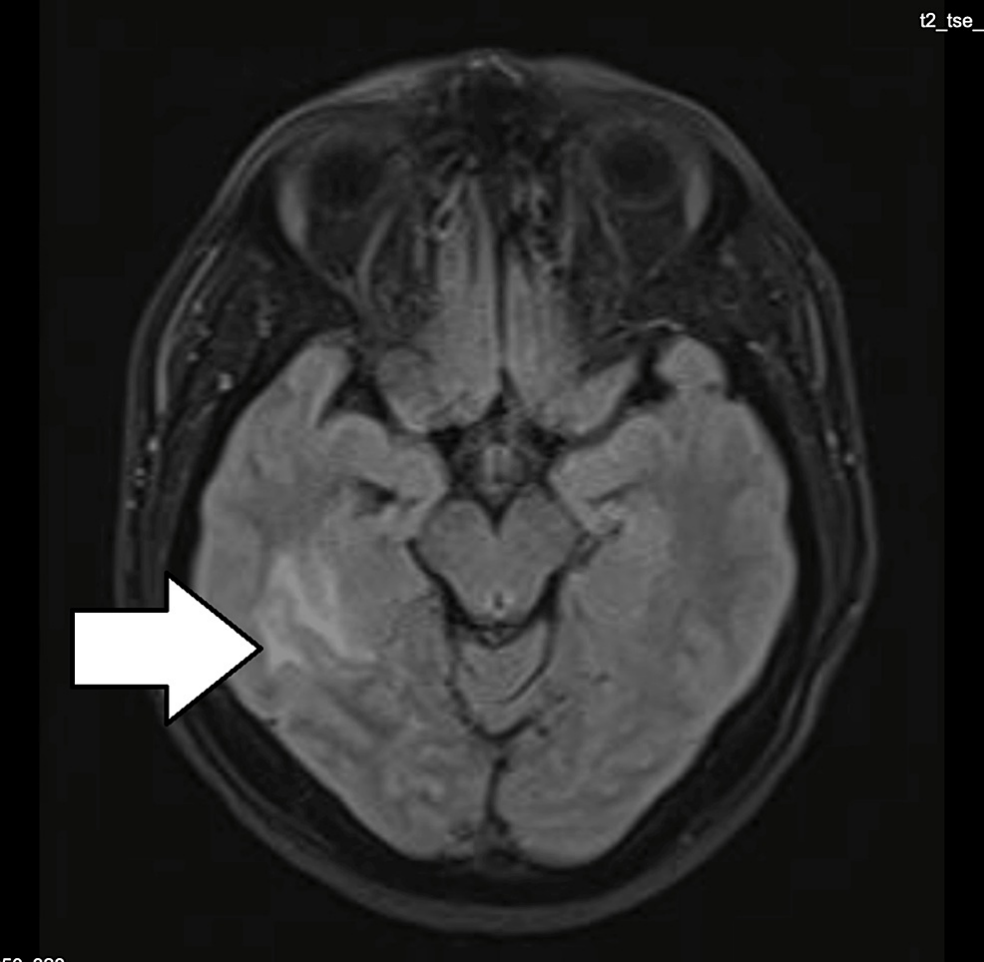
MRI of the brain: T2 flair showing temporal lobe hyperintensity in the right temporal lobe

**Figure 3:**
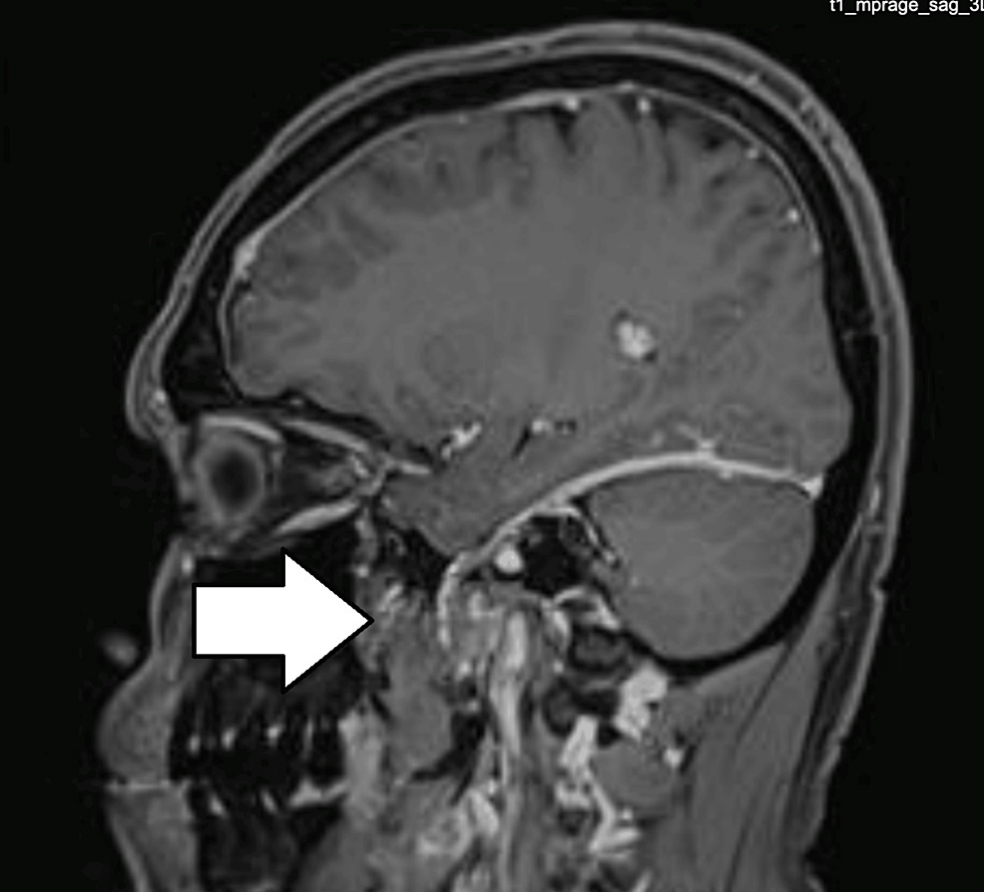
MRI of the brain: T1 contrast, sagittal view, showing contrast enhancement of the meninges

**Figure 4:**
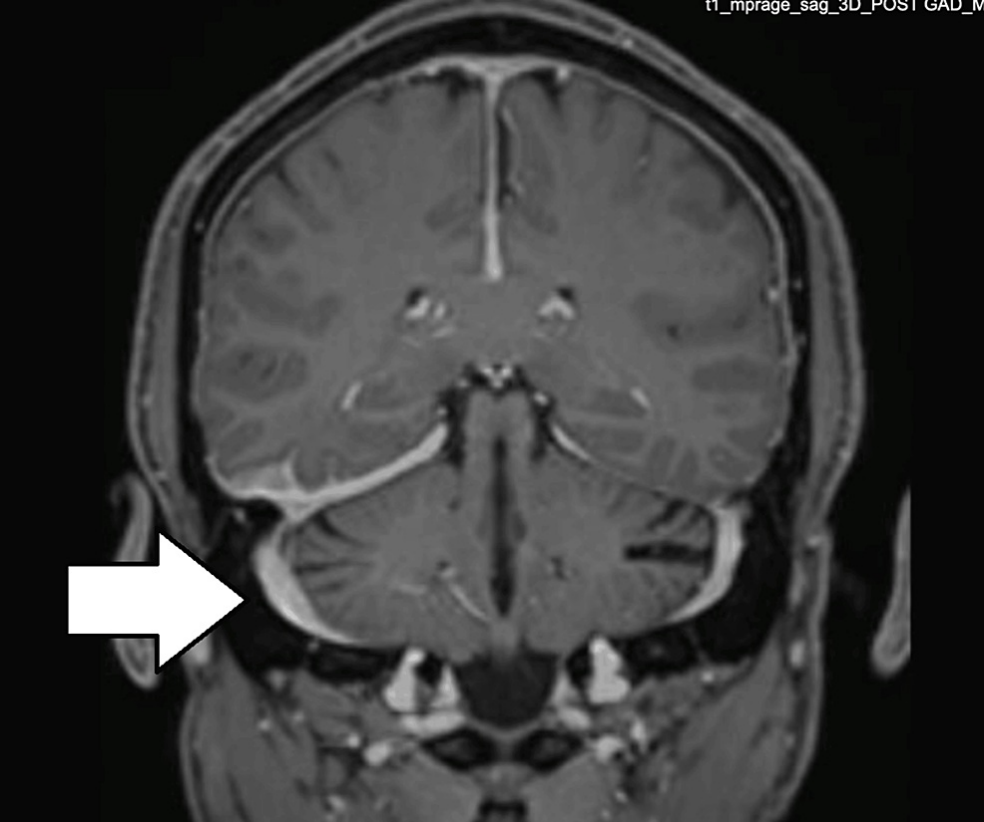
MRI of the brain: T1 sagittal contrast, enhanced view, showing contrast enhancement of the meninges, mainly in the right tentorium cerebellum

His vitamin B12 levels were in the low normal range. Antinuclear antibody (ANA) and angiotensin-converting enzyme (ACE) levels were all within normal limits. The chest X-ray was within normal limits. Cerebrospinal fluid (CSF) analysis revealed elevated proteins of 195.5 mg/dL, suggestive of aseptic meningitis. CSF meningoencephalitis panel and CSF GeneXpert were negative. Brain MRI with contrast revealed diffuse dural thickening and enhancement in both the frontal and parietal convexity and the falx cerebri and along the tentorium cerebelli (right > left); focal leptomeningeal enhancement in the right temporal sulci, suggestive of leptomeningitis; and subtle adjacent flair hyperintensities in the right temporal lobe, suggesting possible early encephalitis (Figures 1-4).

**Figure 5:**
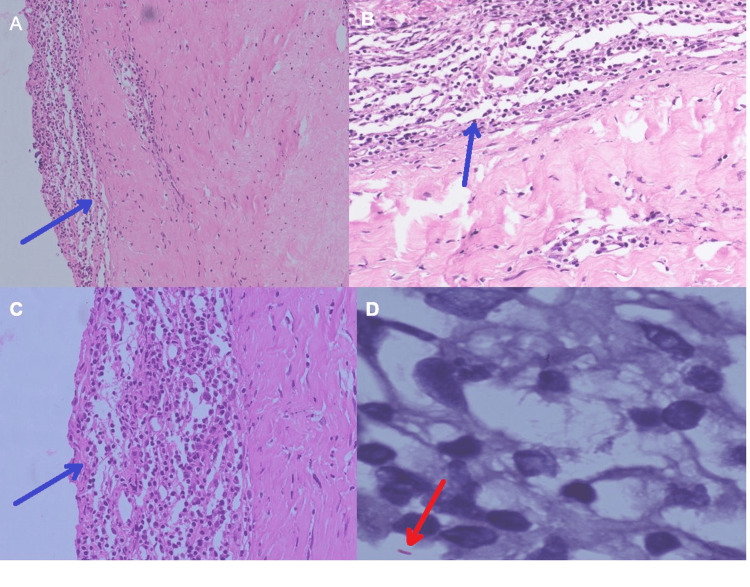
(A-C) Hematoxylin and eosin staining showing thickened dura with lymphocytic infiltration (blue arrows). (D) Acid-fast bacilli in Ziehl-Neelsen stain (red arrow)

Dural biopsy showed features of chronic ill-formed granulomatous inflammatory lesion with occasional acid-fast bacilli, consistent with tubercular pachymeningitis (Figure 5).

Following this, antitubercular therapy for meningitis was started as per the Revised National Tuberculosis Control Program (RNTCP). He was administered intravenous (IV) methylprednisolone 1 g IV q24 for five days and was treated conservatively for his symptoms. He was also given an optineuron injection intramuscularly for his concomitant low-normal B12 levels. The symptoms subsided on medical management with antitubercular therapy, and he was discharged in a hemodynamically stable condition. He was advised tablet prednisolone on discharge with tapering of dose over time.

## Discussion

Hypertrophic pachymeningitis, first described by Charcot and Joffroy, is a rare disorder characterized by the inflammation and thickening of the dura mater, leptomeninges, and tentorium [4,5]. Although the etiologies are diverse, tuberculosis is an important cause of pachymeningitis in developing countries [4,6]. While cranial pachymeningitis typically presents with symptoms of headaches, cranial nerve palsies, focal neurological deficits, and cerebellar ataxia, spinal pachymeningitis presents with symptoms of nerve root compression [6]. Pachymeningitis may also be complicated by intracranial hemorrhage, dural venous sinus occlusion, internal carotid artery occlusion, cerebral edema, Tolosa-Hunt syndrome, and obstructive hydrocephalus [4,7].

A case report by Tariq and Ahmed demonstrates a 46-year-old female who presented with headache and blurred vision [7]. The imaging revealed an extra-axial, dural-based soft tissue mass, which on histopathology revealed granulomatous pachymeningitis suggestive of possible tubercular etiology [7]. The patient responded well to antitubercular therapy [7].

Another case report on tubercular hypertrophic pachymeningitis reveals a seven-year-old female presenting with headache and behavioral disorder with vomiting induced by the insertion of fingers into the mouth, causing undernutrition with cachexia [4]. CSF analysis was negative for bacterial antigens and tuberculosis [4]. Brain MRI showed multifocal nodular abnormalities and meningeal thickening and enhancement [4]. The initiation of anti-bacillary treatment brought great clinical response and weight gain, with the elimination of behavior disorder [4].

There have been reported instances of idiopathic hypertrophic pachymeningitis responsive to antitubercular therapy [8]. Parney et al. reported a 55-year-old female presenting with a right fourth nerve palsy and a chronic history of headaches [8]. MRI revealed thick enhancing dura on the right half of the tentorium cerebelli, with open biopsy, stains, cultures, and chest X-ray negative for acid-fast bacilli [8]. The patient was administered antitubercular therapy given the strongly positive purified protein-derivative skin test and residence in a TB-endemic region [8]. This was followed by improvement in symptoms and signs and the resolution of the tentorial lesion [8].

Cordeiro et al. reported a case of a 62-year-old male who presented with chronic headaches, progressive right-sided vision and hearing loss, and progressive dysphagia [6]. CSF analysis revealed lymphocytic pleocytosis with elevated protein levels, and brain MRI showed dural thickening with biopsy findings consistent with tuberculosis [6]. The patient responded well to medical management with steroids and antitubercular medications [6].

CSF analysis is normal in a quarter of the cases, with elevated protein levels in two-third of the cases and lymphocytic pleocytosis in one-fourth of the cases [4]. Although contrast-enhanced computed tomography (CECT) is used in the diagnosis of dural disease, gadolinium-enhanced MRI is superior to CECT while evaluating leptomeningeal disease [4]. A meningeal biopsy is the diagnostic modality of choice in the case of pachymeningitis when other diagnostic tests are inconclusive [6,7,9]. Since our patient’s CSF analysis and brain MRI were inconclusive, a meningeal biopsy was done, which revealed chronic granulomas and acid-fast bacilli. Due to the nonspecific clinical presentation of tubercular pachymeningitis, delayed diagnosis is often common, resulting in an increased mortality rate [3]. The management of pachymeningitis includes the treatment of the cause and corticosteroids, azathioprine, cyclophosphamide, and immunomodulating agents in the case of resistant diseases [7,9].

## Conclusions

Pachymeningitis is a rare disorder of the dura mater, leptomeninges, and tentorium due to a diverse etiology, with tuberculosis being one of them. The diagnostic challenges associated with tubercular pachymeningitis due to its nonspecific clinical presentation prompt meningeal biopsy in the patients. Although steroids and immunosuppressants are the mainstays in treatment, immunomodulating agents must be considered in refractory cases.

Mycobacterial tuberculosis should always be considered as a possible differential diagnosis while evaluating hypertrophic pachymeningitis, especially when the routine laboratory tests and immunological workup are negative.
